# A biphasic locomotor response to acute unsignaled high temperature exposure in *Drosophila*

**DOI:** 10.1371/journal.pone.0198702

**Published:** 2018-06-08

**Authors:** Daniela Ostrowski, Autoosa Salari, Melissa Zars, Troy Zars

**Affiliations:** Division of Biological Sciences, University of Missouri, Columbia, Missouri, United States of America; Biomedical Sciences Research Center Alexander Fleming, GREECE

## Abstract

Unsignaled stress can have profound effects on animal behavior. While most investigation of stress-effects on behavior follows chronic exposures, less is understood about acute exposures and potential after-effects. We examined walking activity in *Drosophila* following acute exposure to high temperature or electric shock. Compared to initial walking activity, flies first increase walking with exposure to high temperatures then have a strong reduction in activity. These effects are related to the intensity of the high temperature and number of exposures. The reduction in walking activity following high temperature and electric shock exposures survives context changes and lasts at least five hours. Reduction in the function of the biogenic amines octopamine / tyramine and serotonin both strongly blunt the increase in locomotor activity with high temperature exposure. However, neither set of biogenic amines alter the long lasting depression in walking activity after exposure.

## Introduction

Exposure to stressors can have profound effects on animal behavior. While effects of chronic exposure to stress on behavior is relatively well established, including studies in invertebrate animals, much less is known about the effects on behavior of acute unsignaled experiences with aversive stimuli. For example in song sparrows, chronic exposure to food scarcity and high predator pressure has a profound effect on reproduction, causing the nestlings and the broods to be smaller [1]. In rats, long term (3 weeks to 3 months) exposure to different stressors such as water deprivation, food deprivation or exposure to cold temperatures produces changes in sleep and anhedonic behavior [2]. Moreover, in insects like *Drosophila*, extended exposure to aversive heat, electric shock, or vibration impacts multiple behaviors including changes in motivated behaviors like learning, locomotion, and courtship [3–6].

In flies, the decision to walk is fundamental to most behavior. Walking or not walking is intrinsic to grooming and courting, and phases of walking / quiescence reveal circadian rhythms and sleep (e.g., [7–11]). Importantly, this powerful and straight forward indicator of motivated behavior can be modified by stress. For example, *Drosophila* will walk for hours between two visual targets in the Buridan’s paradigm [12, 13]. Addition of chronic exposure to vibration leads to a reduction of walking distance [4]. Moreover, in heat-box and shock-box paradigms, flies will continue to walk back and forth in a dark, narrow chamber for several hours [5, 6, 14]. Exposure to aversive high temperatures or electric shocks over several minutes reduces walking activity and increases escape latencies [3–6]. What is less understood is the acute locomotor response to aversive temperatures or electric shocks.

In our study, we used *Drosophila melanogaster* to measure potential changes in walking activity in response to stress from aversive temperature exposures or electric shock. To accomplish this we used the heat-box apparatus to record walking distance after unsignaled exposures [3, 14, 15]. We presented flies with short exposures of different temperatures or electric shocks and recorded walking distance from minutes to hours after the exposure. Flies exposed even once to high temperature had a biphasic response, with a short increase in activity followed by a decrease, or depression, in walking distance compared to initial walking activity. Electric shock exposure had a similar effect with a decrease in walking distance after exposure (for technical reasons we could not measure walking distance during shock). Warm temperature exposure did not have a noticeable effect on walking distance. A prolonged reduction in walking distance with just a few exposures to high temperatures or shock lasted for 5 to 8 hrs, but returned to control levels within 24 hours. Changes of the biogenic amine systems octopamine / tyramine and serotonin led flies to have a blunted increase in activity with high temperature exposure but did not impact the reduction in walking distance after exposure.

## Materials and methods

### Animals

*Drosophila melanogaster* were raised on cornmeal-based fly food media [16] and maintained on a 12h / 12h day/night cycle at 24°C and 60% relative humidity. For behavioral experiments wild-type Canton S (CS) flies at the age of 2–5 days were used. Males and females were tested separately. Data were similar in both groups and thus combined for presentation here. Animals were briefly cooled down on ice, selected, and then returned to a fly food vial for 1–4 days before testing. Flies were put on a fresh food vial 24 hours before experimentation.

The role of the biogenic amines octopamine / tyramine and serotonin were examined. The TbH[M18] allele on a wild-type Canton-S X-chromosome was used [17, 18]. To alter serotonin signaling, expression of a UAS-driven tetanus toxin light chain (TeTxLC), CYO34-1, was driven in serotonin neurons using the Trh-GAL4 driver [19–21].

### Locomotion assay

Walking activity was measured using the heat-box apparatus [14, 15]. Single flies were placed in a chamber (34 mm x 1 mm x 3 mm), which is lined on one side with a bar-code reader, to monitor the fly’s walking distance over time. Every 100 ms the position of a fly is registered and recorded. The position of a fly varies from 1 arbitrary unit (a.u.), indicating one end of the chamber, to 140, indicating the other end of the chamber. Walking distance [mm] of individual flies was determined based on calibration of the 140 a.u. with the length of the chamber. Up to 15 flies were tested in parallel and walking distance was averaged for each experiment. Walking distance was analyzed under normal conditions (24°C) and after warm to high temperature exposures (29°, 33°, 37° and 41°C). Peltier elements on the top and the bottom of each chamber allowed for fast temperature changes within seconds. Walking distance changes after high-temperature exposure was compared to previous walking distance and presented as normalized walking distance.

To test for transfer of the effect of unsignaled high temperature exposure, flies were exposed to different temperatures outside the heat-box using a plastic tube in a water bath. In this case, flies were first put in the heat-box to record initial walking distance, transferred to the pre-warmed plastic tube for 30 s, and put back in the heat-box to record walking distance.

To test for potential effects of fatigue, flies were immobilized in a pre-warmed plastic tube with drastically reduced space for walking using a sponge stopper. The sponge was placed 1–2 mm above the bottom. Flies were largely immobile during this period.

Electric shock was used as a second potential stressor. Electric shock was delivered to flies through an electrifiable and flexible copper grid that completely lined a plastic tube. Twelve -100 ms shocks were presented to the flies in 5 s intervals for 1 min. Humidity in the environment was controlled and set to 90–100%. Intensity of shocks ranged from 10 to 100 V. As above, flies were first recorded for walking distance, transferred to the shock tube and shocked, and tested for walking distance.

### Data analysis

All experiments were done with N = 8–12 groups of up to n = 15 flies in each group. Thus, n > 120 flies for each group. Inspection of normal values and residuals indicates normal distribution, as is typical for this sort of analysis [22]. Parametric ANOVA and Neuman-Keuls post-hoc tests were used to reveal significant differences. P < 0.05 was considered to be statistically significant.

Data are archived at Dryad: doi:10.5061/dryad.5010rd2.

## Results

**Fig 1:**
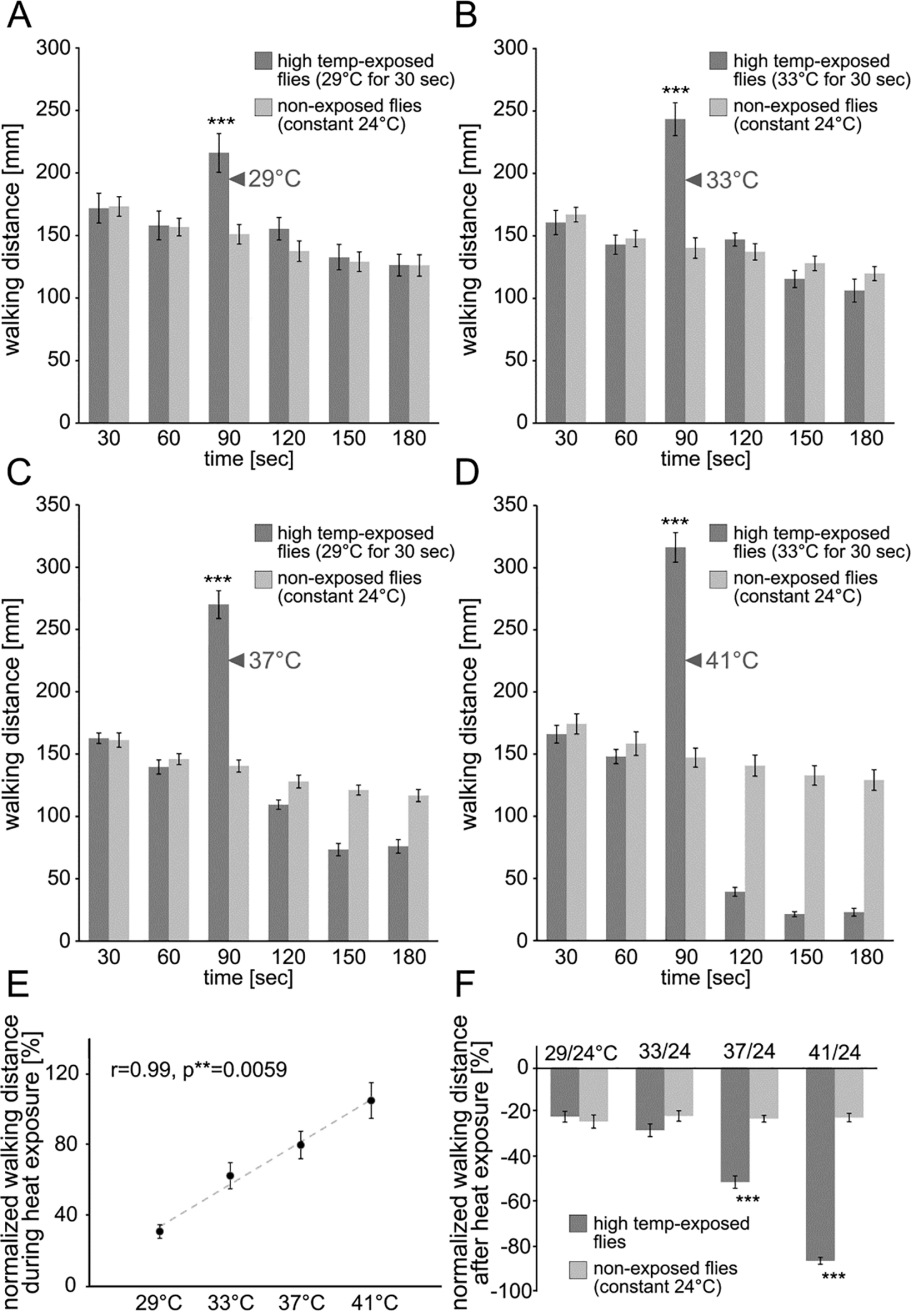
Increase then depression of locomotor activity after high temperature experience. (A-D) Walking distance of flies was recorded over 3 min using the heat-box apparatus. After two initial 30 s periods at 24°C the chambers were heated to 29°, 33°, 37° or 41°C for 30 s (A-D; dark gray columns). After high temperature exposure chambers were cooled down to 24°C. Control flies were kept at constant 24°C (light gray columns). Walking distance increased with high temperature exposure (F = 17.12, P< 0.00001. Newman-Keuls post-hoc tests = p ≤ 0.001 = *** comparing with and without high temperature exposure). (E-F) Normalized walking distance during (E) and after (F) high temperature exposure. During high temperature exposure, fly’s locomotor activity increases. In the range of 29° to 41°C the relationship between temperature and walking distance shows a strong linear correlation (p = 0.0059 = **). After high temperature exposure of 37° and 41°C locomotion is significantly reduced compared to control flies (Newman-Keuls post-hoc tests = p ≤ 0.001 = *** comparing with and without high temperature exposure). The bars represent mean values and the error bars are standard errors of the mean.

Walking distance of *Drosophila* was recorded over a period of 3 minutes using the heat-box apparatus. Individual flies were placed in a small chamber where the position of the flies was detected every 100 ms. Walking activity is presented as total walking distance [mm], binned in 30 s periods (Fig 1A–1D). To record the initial walking activity of the flies, all chambers were kept at 24°C for the first minute of the experiment. A 30 s period of high temperature exposure (29°, 33°, 37° or 41°C) followed. Flies increased walking distance with exposure to high temperatures (Fig 1A–1D). There was a strong linear correlation of the increased locomotor activity for temperatures ranging from 29° to 41°C (Fig 1E). As the probe temperature increased, so did the total walking distance during a 30 s period. After high temperature exposure, chambers were cooled back down to 24°C. Locomotor activity decreased to either ‘normal’ activity, that was not different from control animals (Fig 1A, 1B and 1F) or to ‘depressed’ activity, that was significantly lower than control animals (Fig 1C, 1D and 1F). To examine more closely the properties of depressed locomotor activity, we focused on 37° and 41°C for the remaining temperature experiments.

**Fig 2:**
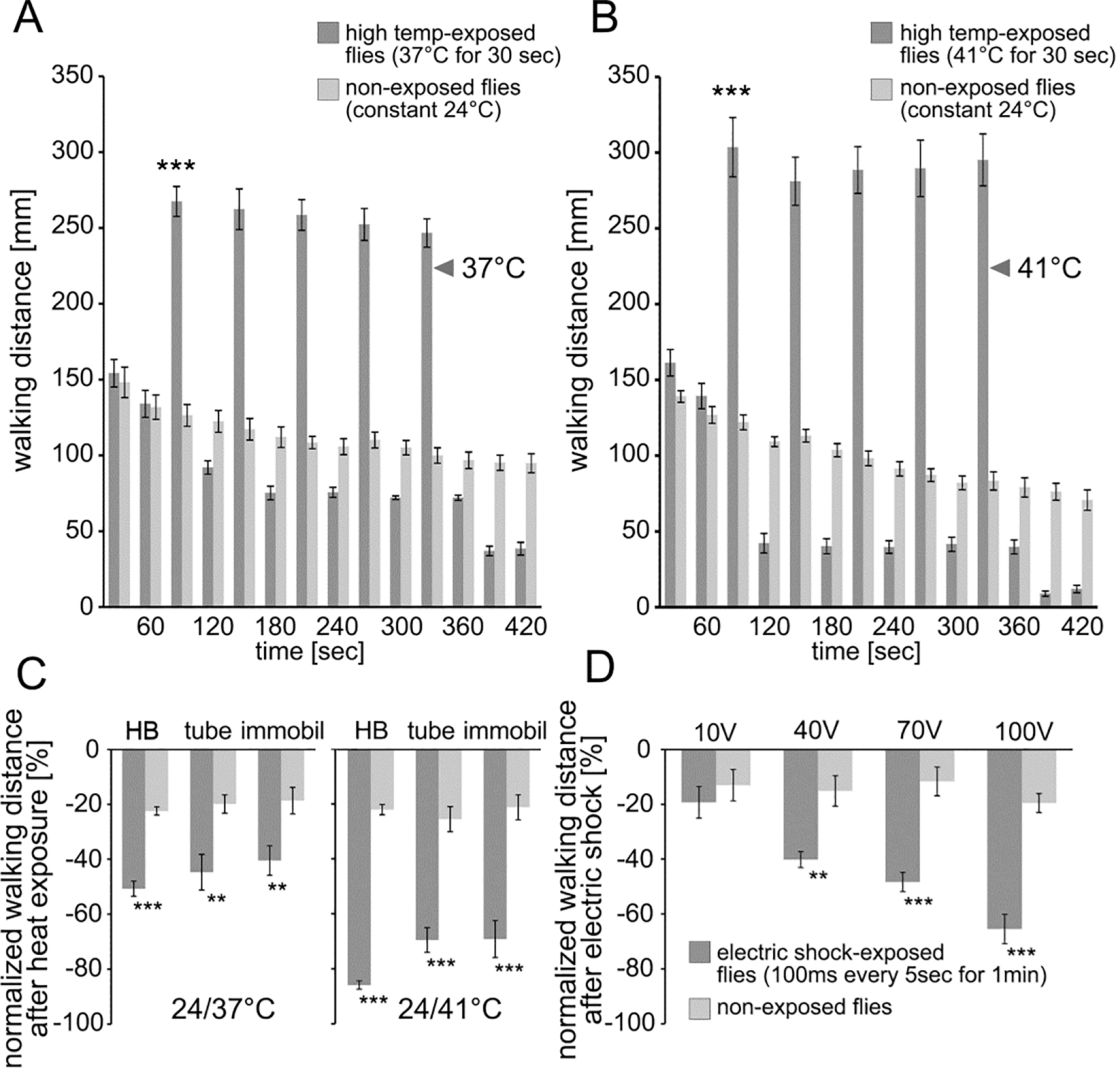
Depressed locomotor activity is not due to fatigue and is a general phenomenon. (A-B) To examine whether or not depressed walking distance is due to fatigue, flies were exposed to high temperature five times. During high temperature exposures walking distance remains high for both 37° (A) and 41°C (B), indicating that flies are still able to maintain a high level of walking (F = 83.8, P < 0.00001, Newman-Keuls post-hoc test between exposed and non-exposed flies p < 0.001 = ***). No statistical difference was detected for walking distance during the five different high temperature exposures (p_37°C_ = 0.68; p_41°C_ = 0.92). (C) Depressed walking distance is context independent and not due to fatigue. Flies were exposed to either high temperatures outside the heat-box (‘tube’) or outside the heat-box with almost no ability to walk (‘immobil’). For both experimental groups and at 37 and 41°C, walking distance decreased similar to flies exposed within the heat-box apparatus (‘HB’ F = 90.1 and 650.7, p_HB_ < 0.001 = ***; ‘tube’ F = 14.6 and 61.9, p_tube_ < 0.01 and < 0.001 = ** and ***, ‘immobil’ F = 20.9 and 40.1, p_immobil_ < 0.01 and < 0.001 = ** and ***). (D) Depressed walking distance was also seen after giving the flies electric shocks as a different aversive stimulus (F = 16.9, P < 0.00001; Newman-Keuls post-hoc comparison for flies with and without shock: p_10V_ = 0.551; p_40V_ = 0.003 = **; p_70V_ < 0.001 = ***; p_100V_ < 0.001 = ***). The bars represent mean values and the error bars are standard errors of the mean.

To test whether depressed locomotor activity was a consequence of fatigue, two experiments were performed. For the first experiment, flies were placed in the heat-box at 24°C for one minute to record initial walking distance. Flies were then exposed to five, 30-s high temperature exposures of 37°C or 41°, each separated by 30 s at 24°C. After the five high temperature exposures, chambers were again cooled down to 24°C. Locomotor activity across each of the five high temperature exposures remained high for both 37°C and 41°C, with no significant change in walking distance between each high temperature exposure (Fig 2A, Fig 2B). However, depressed locomotor activity was still observed upon return to 24°C. For the second experiment, mobility was restricted during heat exposure by placing the flies in an empty pre-heated vial with a sponge stopper directly over the flies, roughly 1–2 mm from the bottom of the vial. The vial was then placed in heated water at control and experimental temperatures. Flies were then placed back into the heat-box at 24°C to record locomotion. As previously seen, walking distance was greatly reduced following exposure to 37°C and 41°C, even under nearly immobilized circumstances during high temperature exposure (Fig 2C). Therefore, exposure to high temperatures decreases locomotor activity whether flies are mobile or not during the 30 s exposure, and does not appear to be a consequence of fatigue.

We also tested whether depressed walking distance is a general response after an aversive experience [22]. Therefore, we analyzed walking activity of flies after electric shock experience (Fig 2D). Flies were first put in the heat-box to record initial walking distance and then collected and transferred to an electric shock tube. The electric shock was delivered to the flies through an electrifiable and flexible copper grid that served as the floor of a small Plexiglas tube. Flies were shocked for 1 minute and then placed back in the heat box to measure locomotor activity. Similar to the results seen after high temperature exposures (Fig 1F), electric shocks of 40 V, 70 V and 100 V significantly decreased flies’ walking activity. Results additionally indicate that the greater the shock intensity the greater the depression in walking activity.

**Fig 3:**
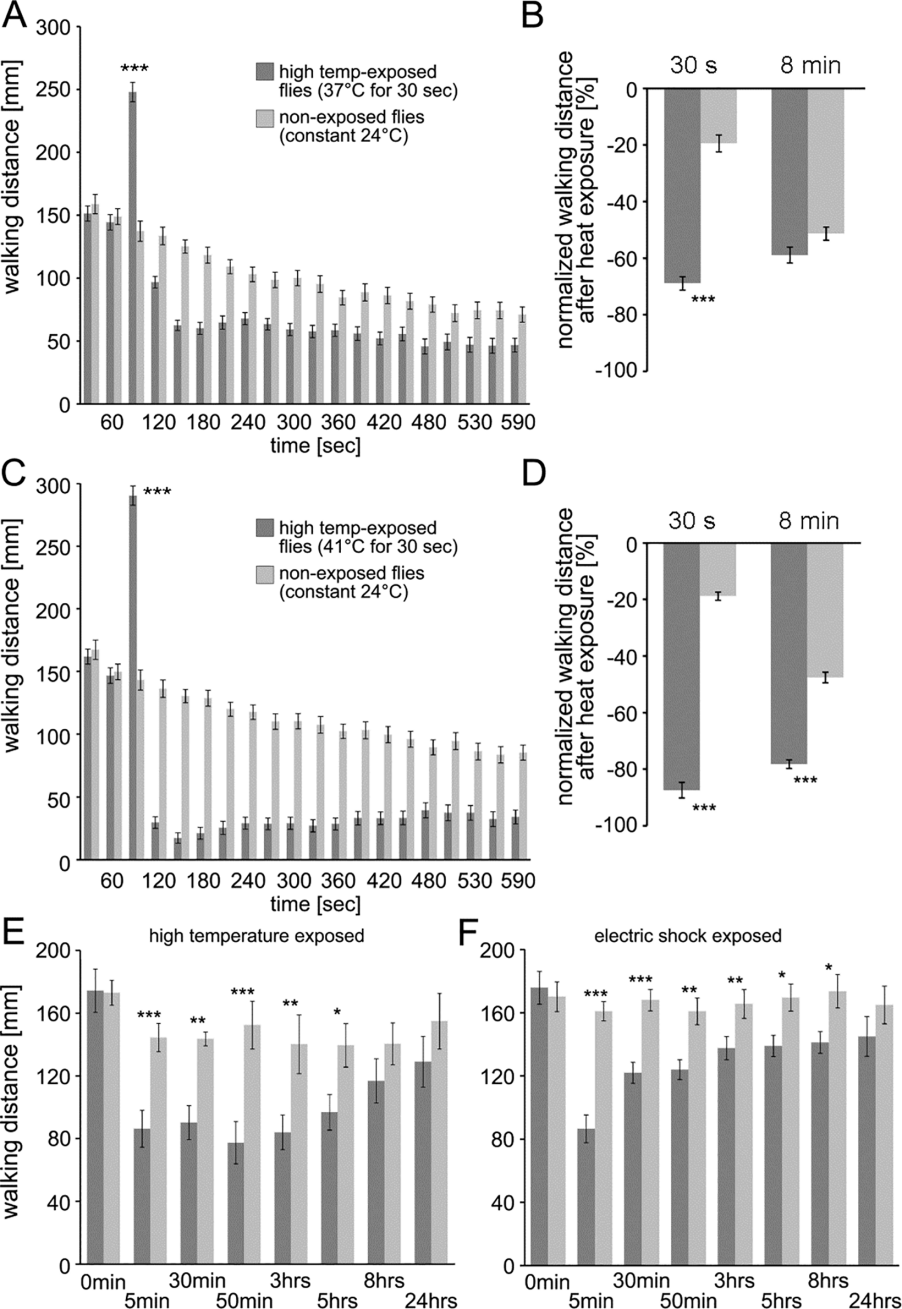
Locomotor depression after high temperature and electric shock exposure is a long lasting change in behavior. (A-D) Extended recording of walking activity after high temperature exposure (37° and 41°C) revealed a steady change in locomotion. Activity increased with high temperature exposure (F = 22.14, P < 0.00001, Newman-Keuls post-hoc test p < 0.001 = ***). Moreover, (B, D) walking activity is strongly reduced 30 s after the 37°C stimulus but largely normal 8 min later (B) and is still highly significantly different for flies exposed to 41°C at both time points (D) when compared to control animals kept at 24°C (F = 33.27 and 81.78, P’s < 0.0001; Newman-Keuls post-hoc test p_37°C 30s_ < 0.001 = ***; p_37°C 8 min_ = 0.47; p_41°C 30s_ <0.001 = ***; p_41°C 8 min_ < 0.0001 = ***). (E,F) Walking distance was depressed after exposure to high temperatures and electric shock. A significant reduction in locomotor activity was evident up to 5 hours after exposure to 41°C, and up to 8 hours after exposure to electric shocks (temperature: Fs’ = 0.007, 15.3, 20.9, 13.6, 7.4, 5.7, 1.5, 1.2. shock: F’s = 0.16, 48.7, 23.8, 12.1, 5.7, 7.9, 6.6, and 1.3. P’s < 0.001 = ***, P’s < 0.01 = **; P < 0.05 = *). The bars represent mean values and the error bars are standard errors of the mean.

Since all experiments thus far recorded walking distance for a total of 3 minutes, we sought to determine how long the observed depressed locomotor effect lasts. We first tested this by extending the recording to 10 minutes and keeping flies in the chambers. For both 37° and 41°C exposure, depressed walking distance remains low throughout the 10 minutes (Fig 3A, Fig 3C). We compared the normalized percent walking distance between high temperature exposed flies and control flies at 30 s (Fig 3B, Fig 3D) and 8 minutes (Fig 3B, Fig 3D) after high temperature exposure. Control flies naturally decrease their locomotor activity over time, therefore just a small difference was detected for flies exposed to 37°C in the long test. However, a strong difference was detected for flies exposed to 41°C at both points. In a second set of experiments, we extended the test series and analyzed changes in walking activity over a time period of up to 24 hrs. Flies were exposed to high temperatures (41°C) several times and kept in fresh food vials between tests. Locomotor activity was recorded at 5, 30, and 50 min and 3, 5, 8 and 24 hrs (Fig 3E). Locomotor activity remained significantly depressed throughout all time points up to 5 hours compared to control flies. For comparison we also analyzed locomotor changes after experience of electric shock (Fig 3F). Locomotor activity also remained significantly depressed up to 8 hours for electric shock exposed flies.

**Fig 4:**
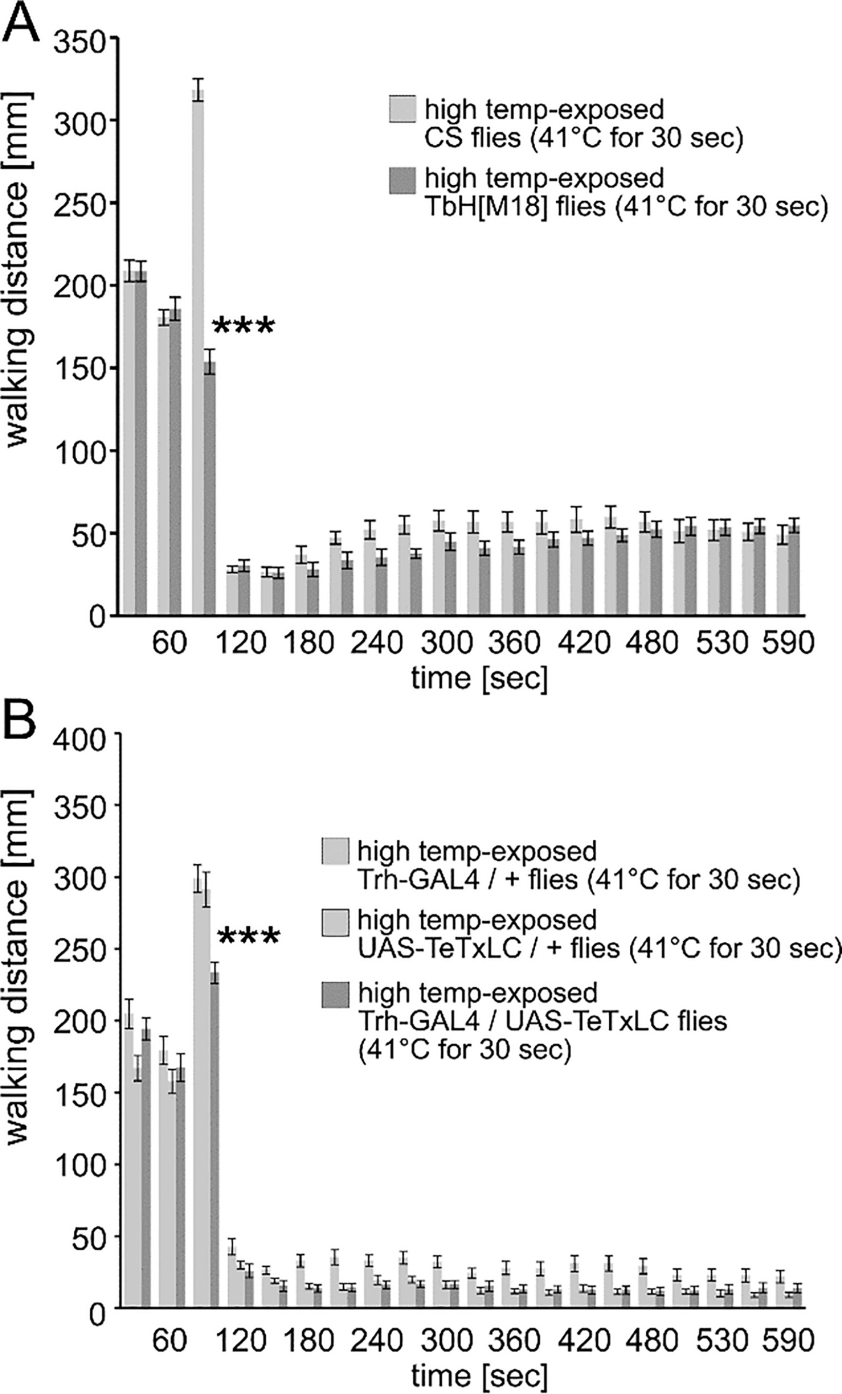
The role of octopamine / Tyramine and serotonin in changes of locomotor activity following high temperature exposure. (A-B) Genetically modified flies that lack the expression of octopamine and increases in tyramine ((A), TbH[M18])) or reduced serotonin function ((B) Trh-GAL4 / UAS-TeTxLC)) were exposed to a single high temperature stimulus (41°C, 30 s) and locomotion was recorded for 8 minutes. (A) The TbH[M18] flies had a strongly reduced response to the 41°C pulse but a similar reduction in walking distance after the exposure (F = 31.01, P < 0.001, Newman-Keuls post-hoc test p < 0.001 = ***). (B) Moreover, Trh-GAL4 / UAS-TeTxLC flies had a reduced initial response to 41°C, but showed a similar reduction in walking distance compared to genetic control flies (F = 2.1, P < 0.02, Newman-Keuls post-hoc comparison p < 0.001 = ***). The bars represent mean values and the error bars are standard errors of the mean.

We examined the potential function of the biogenic amines octopamine / tyramine and serotonin in regulating the changes in locomotor activity with high temperature exposure. In the first test, null mutant tyrosine-beta-hydroxylase (TbH(M18)) flies were tested. TbH(M18) mutant flies have undetectable levels of octopamine and elevated tyramine levels [17, 18]. When TbH(M18) mutant flies were exposed to high temperatures, the response to 41°C was strongly blunted (Fig 4A). The reduction in walking distance after high temperature exposure, however, was nearly identical in wild-type and TbH(M18) mutant flies (Fig 4A). The function of the serotonin neurons in locomotor response to high temperatures was examined by expressing the tetanus toxin light chain (TeTxLC) in these neurons with the Trh-GAL4 driver [19–21]. In this case, the Trh-GAL4 / TeTxLC flies had a reduced response to the high temperature exposure compared to genetic control flies, but a similar reduction in activity afterwards (Fig 4B). Thus, both changes in octopamine / tyramine and serotonin function have an impact on the initial increase of activity with high temperature exposure, but do not alter the after-effect of decreased walking distance.

## Discussion

We show that *Drosophila melanogaster* exhibit a biphasic response to unsignaled high temperature exposure. First there is an increase in walking distance in a given time period with high temperature exposure (Figs 1–4). The increase in walking distance is consistent with increased walking activity in both *Drosophila* and other insects at high temperatures (e.g., [23–25]), and was recently investigated in detail for *Drosophila melanogaster* [26]. Our unique approach, however, allowed us to explore what happens to flies’ locomotor activity, in comparison to initial activity, after the heat exposure interval is terminated. The second behavioral change is a marked decrease in walking distance after an unsignaled high temperature exposure. The first behavioral change, increase in walking distance, is influenced by the intensity of the high temperature exposure, but not the number of exposures (Figs 1 and 2). By contrast the second, decrease in walking distance is influenced by the intensity of high temperature exposures (Figs 1 and 2). Neither the increase in walking distance nor the decrease after high temperature exposure cessation can be explained by fatigue (Fig 2). Flies also showed a lasting change in decreased walking distance which lasted between 5 and 8 hours after exposure. Moreover, exposing flies to unsignaled electric shock, another aversive stimulus, decreased walking distance that lasted at least 8 hours. Finally, tests for the function of biogenic amines in these tests showed that octopamine / tyramine and serotonin both influenced the first rise in activity during exposure, but did not influence the second decreased walking phase.

The role of the biogenic amines octopamine / tyramine and serotonin were investigated in the biphasic activity temperature response. Using the TbH[M18] null allele [17, 27], flies with a lack of octopamine and elevated tyramine levels showed a severely blunted increase in walking distance during high temperature (Fig 4). Intriguingly, the depressed walking activity following removal of the stimulus was completely normal in Tbh[M18] flies. A similar combination of results was evident when serotonin release was blocked with tetanus toxin light chain expression in serotonergic neurons. Again the first response was significantly blunted, but the post-exposure decrease in walking distance was not altered. That both octopamine / tyramine and serotonin have a role in the primary increase in walking activity shows that multiple modulatory systems are involved in this motivated behavior. Moreover, that there is no change in the same altered flies in the post-stimulus decrease in walking activity indicates another system must provide input to a regulator of this walking activity. This could be another modulatory system not covered with the genetic / transgenic tools used here or could be a more direct link from sensory systems to a walking activity regulator.

Unsignaled exposure to aversive stimuli can have profound impacts on behavior. Yoking experiments in the heat-box showed that acute and brief exposures to high temperatures led to reduced walking activity in flies [28]. Moreover, flies in closed loop in yoking experiments had a larger reduction in locomotor activity, suggesting this as an active strategy for place memory formation. Further investigation of acute and limited exposure to high temperature, either 3 one-minute exposures or through yoking experiments with about the same total timing of exposure, leads to *increases* in place memory when flies regained control of the place / temperature contingency [3, 29, 30]. That is, flies exposed to unsignaled high temperatures had a higher place memory when they were later conditioned using normally weak aversive conditions. The higher than expected memory levels after unsignaled exposure is not evident when high temperature or electric shock aversive conditioning is examined [5, 29, 30]. This perhaps reveals a feature that modulation of walking activity by previous unsignaled experience predisposes flies to modulate activity and show persistent place preference to avoid an aversive cue. That the results in the current study show an effect on walking activity for at least five hours suggests that a place memory effect might also last this long.

A depression-like state has been identified in flies exposed to chronic unsignaled exposure to aversive vibration [4]. That is, flies exposed to periods of vibration for three days decrease walking activity and crossing attempts on a catwalk with large gaps. Flies show these effects for at least 14 hours, suggesting a lasting change in motivated behavior. Investigation of 5-HTP and LiCl treatment suggests that exposed flies reach a depression-like state. Although not studied to nearly the same extent, perhaps the acute unsignaled high temperature exposures used in the current study reveals an intermediate stage toward a depression-like state. That the acute exposure effects of high temperature on reducing walking activity do not depend on octopamine / tyramine or serotonin suggests that the mechanisms inducing the intermediate stage and longer lasting depression-like state are different, and only recruited with days of stress inducing experience.

## Conclusions

Flies exposed to high temperatures have a biphasic locomotor response. There is a short lived increase in walking distance followed by a reduction in activity following unsignaled exposure. This reduction in activity is also evident after exposure to high temperatures in a second context and electric shocks. The change in behavioral state lasts between 5 and 8 hours. The octopamine / tyramine and serotonin biogenic amines are critical for the increase in walking distance with high temperature exposures, but reduction in either system has no effect on the post-exposure reduction in activity.
